# Fish and Chips: A Fast Track to Understanding Blood Development

**DOI:** 10.1371/journal.pbio.0030279

**Published:** 2005-07-05

Each day, the bone marrow of an adult makes upwards of 200 billion new red blood cells, along with lesser numbers of white blood cells and platelets. This process, called hematopoiesis, depends on hematopoietic stem cells (HSCs), which divide to make both more stem cells and progenitor cells that differentiate into all the cell types of the blood. The genetic controls on this process are poorly understood. In this issue, Catherine Verfaillie and colleagues show how a two-stage analysis, generation of transcript microarrays followed by functional validation in zebrafish, can identify key regulators of the hematopoietic process.

The study of human hematopoiesis has been hampered in part because it's not possible to use surface markers to identify and isolate HSCs, a technique used to purify other cell types. The authors used a strategy they had previously developed to isolate HSCs from bone marrow and umbilical cord blood that produces a yield up to 10-fold greater than standard protocols for purifying human HSC. Gene expression in this HSC-enriched cell population was then compared with that in an HSC-depleted population using transcript microarrays (“RNA chips”) to identify those genes whose expression was most different between the two groups of cells. They identified 277 genes whose expression in both marrow and cord blood significantly different between the HSC-enriched and -depleted populations. [Fig pbio-0030279-g001]

**Figure pbio-0030279-g001:**
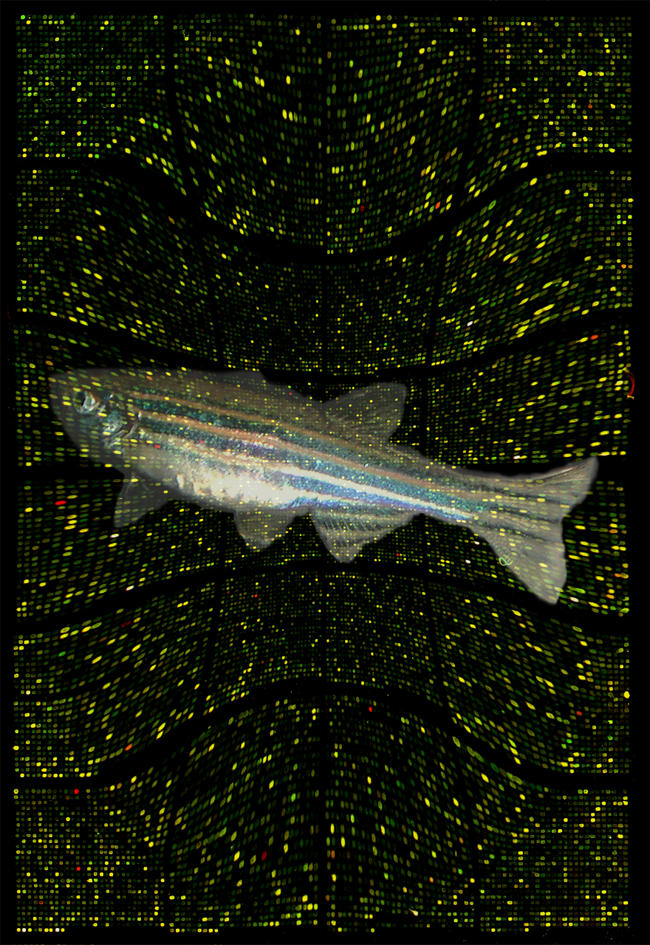
Using a zebrafish microarray, researchers discovered which human genes are active during hematopoiesis, the formation of blood cells

Of these 277 genes, Verfaillie and colleagues identified 61 whose functions were not already known and which had close matches in the zebrafish, a small fish in which hematopoiesis follows essentially the same path as in humans. To prevent expression of these 61genes, they designed complementary antisense molecules against them, and injected them into zebrafish embryos. In 14 of the 61 genes, knocking down expression led to observable defects in hematopoiesis.

The authors note that three of these 14 genes are involved in signaling of fibroblast growth factor, a powerful regulator of development, suggesting that fibroblast growth factor may play a central role in hematopoiesis. More generally, they believe that the combination of using gene transcript microarrays to identify candidates and producing antisense molecules in zebrafish for functional screening of these candidates offers a way to quickly identify genes with central roles in vertebrate development.

